# Inflammatory bowel disease professionals’ attitudes to and experiences of complementary and alternative medicine

**DOI:** 10.1186/1472-6882-13-349

**Published:** 2013-12-10

**Authors:** Annelie Lindberg, Britt Ebbeskog, Per Karlen, Lena Oxelmark

**Affiliations:** 1 Division of Gastroenterology, Department of Clinical Science, Karolinska Institutet, Danderyd Hospital, Stockholm, Sweden; 2 Department of Neurobiology, Care Sciences and Society, Division of Nursing, Karolinska Institutet, Stockholm, Sweden; 3 Institute of Health and Care Sciences, The Sahlgrenska Academy, University of Gothenburg, Gothenburg, Sweden

**Keywords:** Attitudes CAM, Experiences, Healthcare professionals, IBD

## Abstract

**Background:**

Complementary and alternative medicine (CAM) use in patients with IBD is on the increase. Patients report they use CAM when their condition is unresponsive to conventional medication or when they suffer from side-effects, negative stress and disease-related concerns. CAM use may improve patients’ well-being but it can also lead to side-effects and interactions with conventional medications. Research on attitudes to and experiences of CAM among healthcare professionals working with IBD patients is not well studied. Studies in this area could lead to enhanced awareness of and improved communication about CAM between care staff and IBD patients. The aim of this study was to explore IBD professionals’ attitudes to and experience of CAM.

**Methods:**

Sixteen physicians and nurses, 26–70 years old, who had worked with IBD patients for 1–42 years, were recruited. Semi-structured qualitative interviews were conducted. Qualitative content analysis was performed.

**Results:**

Participants stated patients used CAM to improve their well-being generally and there conditions specifically. Participants had a positive attitude towards CAM and respected their patients’ decision to use it, but reported a lack of CAM knowledge. They required education about CAM to be able to meet patients’ needs and provide adequate information. The result of this study indicates that there is a need for CAM education to be implemented in nursing and medical school.

**Conclusions:**

All participants had experience of IBD patients who had used CAM in an attempt to achieve improvement and well-being. Attitudes to CAM were mainly positive, although a problematic aspect was lack of knowledge and evidence in relation to CAM. Implementing CAM education in nursing and medical school will allow healthcare professionals to gain an understanding of therapies widely used by patients with IBD. In clinical practice, using a standard questionnaire regarding CAM use allow healthcare professionals to better understand their patients’ wishes and current CAM use.

## Background

Ulcerative colitis (UC) and Crohn’s disease (CD) are disabling, chronic relapsing inflammatory bowel diseases (IBD). Symptoms are diarrhoea, often with blood or mucus discharge, abdominal pain, weight loss, malabsorption, malnutrition and fatigue. Manifestations from other organ systems such as joints, eyes, skin, liver and bile ducts may also occur. The cause of IBD is unknown and there is no cure, although several therapeutic advances have been made in recent years. The complexity of IBD treatment has increased, and the use of new immunomodulating agents is escalating. The main issue is to combine therapies with minimal side effects and focus on optimising quality of life [1,2]. IBD and its medical treatment have a major impact on patients’ health-related quality of life (HRQOL) [3-5], which may be the reason for their interest in complementary and alternative medicine (CAM) [6].

The World Health Organization (WHO) defines CAM as a broad range of healthcare practices that are not part of a country’s tradition and not integrated into the main healthcare system [7]. CAM can be categorised as alternative medical systems, mind-body interventions, biologically based therapies, manipulative and body-based methods and energy therapies [8]. Complementary medicine refers to the use of CAM in combination with conventional medicine, while alternative medicine implies the use of CAM instead of conventional medicine. Integrative medicine combines both conventional and evidence based CAM treatment [8].

People are willing to spend a great deal of money on CAM treatment [9,10]. In a review Harris et al. [11] showed that CAM was frequently used by the general population in 15 countries. In fact the prevalence of all types of CAM use ranged from 9% to 76%. CAM use has increased in Denmark, Norway and Sweden from 22%-33% to 34%-49% [12]. In Norway and Denmark, half and one third of hospitals respectively offer CAM treatments to patients [13]. It is well known that patients with chronic diseases use CAM [14,15], including IBD [16-19]. In one study conducted at an IBD centre in Stockholm, Sweden, CAM use was 32% [20].

There are various reasons why IBD patients turn to CAM, e.g. they may be unresponsive to or suffer side-effects from conventional treatment or have heard/read that CAM can be helpful [21]. Individuals suffering from IBD want to regain control over their illness [22] and appreciate the fact that CAM focuses on the whole person [19,23]. Intensive long-term corticosteroid therapy [24], negative stress and disease-related concerns [23] have been found to correlate with CAM use among IBD patients. Patients do not tell their physician about using CAM if the physicians do not suggest it [25] and it has been found that the decision to inform the physician about CAM use depends on the quality of the patient-physician relationship [21].

CAM treatments are not without risks, for example, CAM can interact with conventional medication [26]. CAM treatments such as noni juice (Morinda citrifolia) [27,28] or other herbal medicinal products can be toxic to the liver [29], which is important to know, given some IBDs may be associated with disorders in the liver and biliary tract [30-32]. Thus, knowledge of patients’ use of CAM, will allow healthcare professionals to better monitor patients and their responses to treatments.

In 2006, an EU directive on “traditional herbal medicinal products” was incorporated into Swedish legislation. Coupled with the fact that patients with chronic diseases use CAM, this suggests healthcare professionals to be knowledgeable about such products [33]. Healthcare in Sweden is regulated by the Patient Safety Act [34], which sets out the duties and responsibilities of healthcare professionals. A healthcare professional is defined as a person with a health qualification or a protected occupational title who provides care in a hospital or clinic. CAM practitioners are not considered healthcare professionals in Sweden [34].

Research on IBD healthcare professionals’ attitudes to and experience of CAM is not well studied in Sweden, so far. However in one US study, physicians expressed sympathy towards IBD patients’ use of CAM because of IBD’s problematic etiology and management options [35], by highlighting IBD healthcare professionals’ attitudes to and experience of CAM, problem areas ought to be identified. Experience is a philosophical concept and something we constantly perceive in continuous interaction with the environment [36]. It is important to reflect on the meaning of the experience in what you do, what happens and how this affects your attitude. Healthcare professionals’ attitude plays an important role in the establishment of the relationship with the patient and healthcare professionals are responsible for their attitudinal reaction [37]. Increased awareness and knowledge of CAM are necessary in order to enhance communication in IBD professionals’ encounters with their patients and thereby the quality of care. The aim of this study was to explore IBD professionals’ attitudes to and experience of CAM.

## Methods

The design was a qualitative study with semi-structured interviews. A qualitative perspective, content analysis, was used to create an understanding of the phenomenon IBD professionals’ attitudes to and experience of CAM [38,39].

### Participants

The respondents were RNs and physicians recruited from four IBD clinics in two metropolitan areas. Purposeful sampling was used to obtain as much variation as possible in terms of experience and to achieve insight into the phenomenon, IBD professionals’ attitudes to and experience of CAM [39]. The variables on which purposeful sampling were based on was profession, gender, age and years in IBD care.

### Data collection

RNs and physicians were contacted directly at the IBD clinics. Sixteen healthcare professionals were recruited in accordance with the sampling framework (15–20 persons). All those invited agreed to participate. The interviews were conducted using a semi-structured interview guide with open-ended questions to fulfil the aim of the study [39]. Participants were asked about their attitudes to and experience of CAM according to their own perspective. No definition of CAM was given before the interviews. Supportive questions were added during the interview (What do you mean? Could you explain?) A test interview was performed to assess the validity of the interview guide [39]. An audit trial of this interview was conducted by an external research group, which confirmed credibility. The first author (AL), who has 17 years of experience as an IBD nurse, performed all interviews with the exception of two, which were conducted by the last author (LO) to ensure credibility and avoid bias [40]. The interviews were conducted in a separate room, and took place at the participants’ IBD clinics, each of which lasted from 15–50 minutes. Before the interview the participants gave their informed consent.

### Data analysis

Content analysis is a research technique for analysing relatively unstructured data in order to arrive at replicable and valid conclusions from texts in their context [38,39]. Krippendorff’s manifest qualitative content analysis method was used, as it is appropriate for analysing text that focuses on communication and experiences, which is in line with the aim of the study. A core feature of qualitative content analysis is the development of categories on the basis of similarities and differences at various logical levels [38]. The interviews were recorded and transcribed verbatim. In the first step, the authors read the entire text several times in order to become familiar with it and gain a sense of the whole. Two domains were recognized: description of attitudes and description of experiences. In the next step, meaning units corresponding to the two domains were extracted and coded. Sentences and phrases containing information relevant to the aim were understood as meaning units. Each meaning unit was condensed, labelled, organised into groups and coded according to domain. This was followed by classifying and abstracting the meaning units into sub-categories and categories to highlight the content. During the analysis efforts were made to remain close to the interview text. Sub-categories and categories were identified by moving back and forth between categories, meaning units, domain**s** and text. Data were analysed by the researchers and the outcomes discussed to ensure reliability. Disagreements were debated until consensus was reached. The researchers jointly discussed the validity to ensure that the sub-categories and categories were mutually exclusive and exhaustive [39,40].

### Ethical consideration

The study was approved by the Regional Ethical Review Board in Stockholm. All participants were assured of confidentiality, informed that participation was voluntary and that they could withdraw at any time.

## Results

Sixteen IBD professionals (seven physicians and nine nurses) were interviewed. Their mean age was 49 years (range 26–70), 11 were women and five were men. Their mean year of clinical experience in the area of IBD was 20 years (range 1–42). The results are presented in two domains; attitudes and experiences and each domain generated four categories. Each category had two to six sub-categories (Table 1).

**Table 1:** Overview of domains, categories and subcategories

**Domains**	**Categories**	**Subcategories**
**Attitudes**	Creating trust and a good relationship with the patient	Strategies for the encounter with Patients
		Searching for information on patients’ behalf
		A positive attitude to CAM
	Wishing for knowledge and understanding	Willingness to learn about CAM
		Searching for relevant education
		Raising the level of awareness about and concretizing patients’ CAM use
	Trust in CAM	Acceptance of CAM
		CAM as a complement to conventional treatment
		Untapped resource
		Right time for CAM
		Confidence in manual therapies
	Attitudes that constitute a barrier	Restrictive approach
		Distanced view
		No need of or interest in CAM
		Personal definition of CAM
		Reticence about CAM
**Experiences**	Motives for CAM use	Concern about side-effects of conventional medication
		Patients’ wish to avoid conventional medication
		Disease course
		Interest
		Influences from the surrounding environment
		CAM provides a holistic perspective
	Perceiving patients’ quest for improvement and well-being	The patient is searching
		The patient believes in the efficacy of CAM
		The patient informs about her/his CAM use
	Problematic aspects	Limited knowledge/education
		Safety and efficacy
	Differing CAM experiences	Personal experience of CAM
		Influence of pati ents’ narratives about CAM
		Experiences of friends/relatives
		Professional experience of CAM

## Attitudes

### Category I. Creating trust and a good relationship with the patient

This category included three sub-categories; strategies for the encounter with patients, searching for information on the patients’ behalf and a positive attitude to CAM.

The IBD professionals considered that honesty, respect, creating trust and adopting an open attitude were important strategies when encountering patients, for example, having a dialogue with the patient, as expressed by one of the participants:

*I say to my patients, you can take what you want as long as you informme and that it doesn’t make you worse in any way. (No. 3)*

The informants emphasised the importance of treating patients with respect when they informed about their CAM use. It was also essential to adopt a respectful attitude in relation to patients’ decision to use CAM in order to create a trusting relationship.

You have to be very careful not to have a superior attitude towards the patient and not say that it’s nonsense and to stop taking it. That would be very dangerous and can ruin the relationship with the patient. (No. 2)

The healthcare professionals stressed the importance of creating trust and a good relationship with the patients as well as maintaining contact and assisting her/him in searching for information about CAM. Several expressed a positive attitude towards CAM in addition to curiosity about and an interest in different CAM methods.

### Category II. Wishing for knowledge and understanding

This category generated the sub-categories; willingness to learn about CAM, searching for relevant education and raising the level of awareness about and concretizing patients’ CAM use.

It was clear that the participants wanted to learn more about CAM. They stated that it would be interesting as well as stimulating to learn more and be able to support patients when they had questions about CAM treatment.

When considering our patients and their problems, it would be very interesting to learn more. (No. 14)

The informants expressed a wish for relevant education, mainly in the form of lectures and dialogue rather than written information via e-mail or letter. They stated that the knowledge transfer should be mediated by a person who provides information about CAM in an objective manner based on competence in the area. The IBD professionals wanted factual and well founded knowledge that they could communicate to their patients.

I don’t believe in mailshots, you should go and listen to someone who provides the information in an objective way, not some fanatic who is sold on something. (No. 2)

The informants considered it valuable to be told about the patients’ use of CAM in order to make them aware of and concretise their CAM use. They exemplified this by saying that it was important to pose a direct question to the patients about whether or not they used CAM. Some did not routinely pose such questions and merely asked about herbal remedies, while others only questioned patients with liver diseases, as it could be caused by such products.

I usually put questions to patients with liver disease, but rarely to those with IBD. (No. 12)

### Category III. Trust in CAM

This category generated the sub-categories; acceptance of CAM, CAM as a complement to conventional treatment, untapped resource, right time for CAM and confidence in manual therapies.

The level of acceptance of CAM varied among IBD professionals and became higher when CAM was used as a complement to conventional medication.

I suppose it’s okay if it’s used as a complement to ordinary healthcare. (No. 4)

CAM was considered an untapped resource that had a place within healthcare. Some participants were of the opinion that conventional healthcare was inadequate, arguing that CAM fulfilled a function from a psychological perspective. Others claimed that interest in CAM had increased in society and that it was the right time for it. Some expressed confidence in manual therapies, as they believed that touch could induce well-being in patients.

I definitely believe in touch, if you feel taken care of, you will feel better. (No. 5)

### Category IV. Attitudes that constitute a barrier

This category generated the following sub-categories; restrictive approach, distanced view, no need of or interest in CAM, personal definition of CAM and reticence about CAM.

The IBD professionals described a restrictive approach to CAM, expressing concern that patients had too much confidence in herbal remedies. They feared that CAM would become more popular than conventional medication, as expressed by one participant:

I’m a bit concerned about the risk involved in using CAM in place of traditional medication. (No. 4)

Several informants had a distanced view of CAM, being uncertain about what attitude and approach to adopt and tried to remain neutral. Only a few were sceptical although many had a restrictive attitude to herbal remedies and over-the-counter products due to experiences of side-effects. These IBD professionals considered that CAM was unnecessary, due to the number of effective medications available for IBD treatment. Few believed that CAM could help patients with IBD and only one informant expressed a specific interest in CAM. The IBD professionals found it difficult to define the concept of CAM and one participant claimed that the terms complementary and alternative were synonymous. The concept was regarded as diffuse and some were of the opinion that CAM is not provided in today’s healthcare system.

I haven’t grasped whether CAM differs from or belongs to alternative medicine. (No. 1)

The participants perceived that patients were reluctant to talk about CAM and did not spontaneously inform them that they used such methods. Others believed that the reason was that patients were afraid that this information would be treated with contempt.

I think they don’t want to make a fool of themselves in front of the doctor by relating that they use CAM. (No. 4)

## Experiences

### Category I. Motives for CAM use

This category generated the sub-categories; concern about side-effects of conventional medication, patients’ wish to avoid conventional medication, disease course, interest, influences from the surrounding environment and CAM provides a holistic perspective.

Several of the informants stated that an important reason for IBD patients’ CAM use was that they worried about the side-effects of conventional medication and therefore wished to avoid such medication.

I think that many IBD patients are afraid of the side-effects of conventional medicines. (No. 2)

The participants reported that in many cases long-term disease and complicated disease course were the reasons these patients tried CAM methods. They also perceived that patients with a chronic condition more often tried CAM to ensure that they had done everything possible in order to regain health. In their view, some of these patients were interested in CAM, which influenced them to start using it.

I think that a lot of them are very open, especially those who are ill as they try out all avenues in order to regain their health. (No. 6)

However, the IBD professionals also stated that CAM use decreased when conventional treatment was satisfactory. They perceived that CAM was more important during remission than during an acute flare. The respondents considered that the surrounding environment such as the media and financial aspects influenced CAM use. They held that the media could delude patients into believing in CAM methods that had not been evaluated and emphasised that such methods were not subsidised by the State. Some took the view that younger patients seemed better informed about and more familiar with CAM methods compared to older ones. Other factors perceived to influence CAM use were traditional and cultural differences. They commented that in comparison to Sweden, CAM was more easily accessible in other countries and that physicians educated abroad contributed knowledge about CAM:

Half of the doctors who are qualified today were educated outside Sweden and of course they will contribute a great deal. (No. 4)

Some informants reported that they lacked the holistic perspective that takes account of patients’ perceptions and that characterises CAM, something that was missing in traditional healthcare. One respondent commented:

CAM embodies much that has been lost in patient care today, namely care of the whole patient. (No.1)

### Category II. Perceiving patients’ quest for improvement and well-being

This category generated the following sub-categories: the patient is searching, the patient believes in the efficacy of CAM and the patient informs about her/his CAM use.

The IBD professionals perceived that many patients expressed a specific interest in and asked for CAM treatment. They believed that patients did everything in their power to regain health and would try CAM sooner or later. They also stated that patients were very interested in CAM*,* believed in its efficacy and frequently asked health professionals about it.

I think they are well aware that the drugs we provide are quite strong and want to find an alternative, thus they very often come and ask, is it okay to try this? (No. 7)

The participants perceived that the IBD patient group was active in society, highly educated and ambitious. The professionals’ views varied in terms of whether or not the patient told them about CAM use. However, the majority perceived the patients as honest and informing them, while some believed they were not truthful due to fear of being disparaged by the doctor or because they had forgotten to inform about their CAM use. Furthermore, they perceived that the patients had too much trust in CAM, believing it to be harmless and without side-effects.

Many patients believe that herbal remedies are something natural, not real medications, and good for the body and non-toxic, which I can understand. (No. 4)

### Category III. Problematic aspects

This category generated the sub-categories; limited knowledge/education and safety and efficacy.

The IBD professionals found it difficult to obtain knowledge about CAM due to the large number of CAM methods and the fact that CAM was not included in their basic education. One informant was of the opinion that no CAM education was necessary. However, the majority reported that they lacked knowledge about CAM. CAM was perceived as a limited area and the informants would like impartial information about safety and data on efficacy. They stressed the need for a system to control CAM methods and the importance of designing randomised control studies. One important aspect mentioned by several of the informants was that CAM methods must not interact with patients’ ordinary medication for treatment of an acute onset.

CAM must not interfere, interact with essential acute treatment or delay a treatment of an underlying or a diagnosed illness. (No. 1)

### Category IV. Differing CAM experiences

This category generated the sub-categories; personal experiences of CAM, influence of patients’ narratives about CAM, experiences of friends/relatives and professional experiences of CAM.

The informants had varying experiences of CAM. Many had a positive personal experience of manual therapies and others had relatives who had tried CAM.

I personally have positive experiences of massage and zone therapy. (No. 6)

A number of the informants had no personal experience of CAM. However, all had experienced patients using different CAM methods with both positive and negative outcomes.

I have met many over the years who attended the anthroposophic clinic. (No. 2)

## Discussion

This study was conducted to explore IBD professionals’ attitudes to and experience of CAM. The main findings reveal that IBD RNs and physicians had trust in and a positive attitude to CAM, especially when used as a complement to conventional medicine. The IBD professionals’ reported that patients requested and tested various CAM methods. Despite the fact that CAM practitioners are not considered healthcare professionals in Sweden [34], the participants stated that CAM belonged within healthcare, and was relevant to conventional medical care. However, they reported attitudes that constituted a barrier, exhibited a restrictive approach and considered CAM unnecessary, and a few were sceptical. Despite their trust in CAM, only one participant used CAM on a daily basis in clinical practice. Lack of congruence between attitudes and clinical practice has previously been reported by McDowell et al. [41]. One reason may be the participants’ perceived lack of CAM knowledge and education, which underlines the need for further CAM education and intervention studies in clinical settings. Several of the participants reported that they wished for knowledge and understanding about CAM, which is in line with Wong et al. [42]. A German study revealed that the majority of the participating GPs integrated one or more CAM therapies in their everyday clinical practice in primary healthcare [43]. In a previous review of healthcare professionals’ attitudes towards CAM, physicians were less positive compared to other professional categories [44]. Although the objective of the present study was not to assess differences between physicians and RNs, nevertheless no differences in their attitudes or experiences were identified.

The IBD professionals also described problematic aspects and expressed a critical attitude towards herbal remedies, mainly due to lack of knowledge and the risk of side-effects. None of the participants had any particular knowledge of the EU directive concerning “traditional herbal medicines” introduced in Sweden in 2006 [33]. Healthcare professionals should be aware of the potential benefits and risks of herbal medications in addition to their interaction with conventional medication. It is mandatory for physicians and RNs to report all new or serious suspected side-effects of conventional pharmaceutical drugs, as well as herbal remedies, to the Swedish MPA. A review of the reports (n = 64 493) made to the National Swedish Pharmacovigilance system between 1987 and 2006 revealed that 967 concerned suspected adverse reactions related to 175 different CAM products, some of which were unknown and poorly documented. Considering the wide use of CAM, the number of reports was fairly low. Encouraging and facilitating all consumers to report adverse events reactions should improve CAM safety monitoring [45]. The IBD professionals in the present study stated that a problematic aspect of CAM was the lack of regulatory framework. This problem has been addressed by pointing out the gap between CAM policies and practices in Sweden and calling for policy initiatives and dialogue [46].

The lack of CAM knowledge and education expressed by the participants is another problematic aspect. Previous research among Swedish registered professions in surgical care has demonstrated similar results [47]. IBD professionals had experienced patients who had tried various CAM methods and pointed out the importance of guiding the patients. However, the vast majority considered themselves unqualified or lacking sufficient knowledge of CAM to provide appropriate advice and recommendations. In the present study, the respondents expressed a wish for knowledge and understanding and were interested in learning more about CAM modalities. This indicates the need for CAM education for healthcare professionals.

IBD professionals considered it vital to create trust and a good relationship with the patient and for this reason some did not ask about CAM, as they feared that such a question could have a negative influence. A French survey revealed that 75% of IBD patients never discussed CAM use with their IBD physicians, one reason being a poor patient-physician relationship [21]. Richardson et al. [48] asked patients diagnosed with cancer and their physicians about the reasons for nondisclosure of CAM use and found that both groups disagreed significantly on every reason for nondisclosure. In response to the statement “Physicians would discourage or disapprove CAM use”, 80% of the doctors answered that this may be a reason why patients do not reveal such use, while only 13% of the patients agreed with this statement. On the other hand, 47% of the patients stated that one reason for not reporting CAM use was that physicians never asked [48]. However, most of the respondents in the present study believed that the majority of patients were honest about their CAM use. To further assess these issues interviews with IBD patients has now been conducted by our study group. To avoid misunderstandings, it is important for healthcare professionals to invite patients to an open dialogue and not criticise their patient’s decisions. What is considered unconventional therapy today may very well be viewed as conventional in the future.

In the present study the IBD professionals experienced patients’ quest for improvement and well-being by means of various CAM methods. It is essential to explore healthcare professionals’ attitudes to and experiences of CAM to help them better respond to their patients’ needs and wishes. The ultimate goal of healthcare is to provide quality care and improve patients’ health. Awareness of, increased knowledge and education about CAM and its implications, in addition to an understanding of how IBD patients and healthcare providers can use CAM, will facilitate healthcare professionals to achieve this goal. The results of this study indicate that there is a need for CAM education to be implemented in nursing and medical school. A standard questionnaire regarding patients CAM use may facilitate a dialogue between healthcare professionals and patients about CAM, including their CAM use.

### Methodological considerations

The participants in the present study were from the same context, i.e., care of IBD patients, and had varying attitudes to and experience of CAM. Generalisability of the result is not a goal of qualitative research. Qualitative studies are often judged on the basis of trustworthiness, which concerns transferability, credibility, confirmability and dependability [49]. With regard to transferability, these findings may be applicable to other care givers in a similar context. Transferability was enhanced by many rich descriptions of the phenomenon. Credibility was strengthened by choosing informants of different ages, genders and with varying professional IBD experience. Confirmability was ensured by interviewing physicians and RNs from two different urban areas. To strengthen and enrich the analysis, three of the researchers (AL, BE, LO) coded, discussed and followed up the analysis until consensus was obtained [50]. Dependability was achieved by becoming familiar with the data and continuously returning to the original data to ensure that the emerging categories and sub-categories took account of all relevant aspects [38].

## Conclusion

In Sweden IBD professionals had experienced that patients sought improvement and well-being by using CAM. The participants had a positive attitude to and respected their patients’ decision to use CAM. However, problematic aspects were lack of knowledge and evidence in relation to CAM. IBD professionals required CAM education to be able to meet patients’ needs and provide evidence based knowledge. Implementing CAM education in nursing and medical schools will allow healthcare professionals to gain an understanding of therapies widely used by patients with IBD. The results demonstrate that IBD professionals consider CAM an asset, but mainly as a complement to conventional medical treatment. Participants emphasised the importance of dialogue with the patients as well as openness to patients’ interest in and wish to use different CAM methods. What is considered unconventional therapy today may very well be viewed as conventional in the future.

## Competing interests

All authors declared that they have no competing interests.

## Authors’ contributions

All authors participated in the conception and design of the study. AL has collected the data, transcribed the interviews analysed the data, written and revised the manuscript. LO performed two interviews and transcribed one interview. AL, BE and LO, interpreted the data, coded, discussed and followed up the analysis until consensus was obtained. BE, PK and LO has critically revised the manuscript. All authors have read and approved the final manuscript.

## Pre-publication history

The pre-publication history for this paper can be accessed here:

http://www.biomedcentral.com/1472-6882/13/349/prepub
